# Subject-Independent Depression Recognition from EEG Using an Improved Bidirectional LSTM with Dynamic Vector Routing

**DOI:** 10.3390/bioengineering13030358

**Published:** 2026-03-19

**Authors:** Ziqi Ji, Kunye Liu, Weikai Ma, Xiaolin Ning, Yang Gao

**Affiliations:** 1 School of Instrumentation Science and Optoelectronic Engineering, Beihang University, Beijing 100191, China; 2 Hangzhou Institute of National Extremely-Weak Magnetic Field Infrastructure, Hangzhou 310051, China; 3 Hefei National Laboratory, Hefei 230088, China

**Keywords:** depression diagnosis, electroencephalographic (EEG) signals, multi-domain fusion, bidirectional LSTM, deep learning

## Abstract

Electroencephalography (EEG) has become an increasingly important tool in depression research due to its ability to capture objective neurophysiological abnormalities associated with depressive disorders, offering high temporal resolution, non-invasiveness, and cost-effectiveness.However, existing methods often fail to fully exploit the multi-domain information in EEG signals, resulting in limited model generalization capabilities. This paper proposes an improved bidirectional long short-term memory (BiLSTM) model that segments continuous EEG into non-overlapping 2-s epochs and learns end-to-end from multi-channel temporal sequences. After band-pass filtering and resampling, each epoch is represented as a channel–time matrix $X\in R^{C\times T}$ (with C = 128) and processed by a BiLSTM encoder followed by a dynamic-routing encapsulated-vector classifier. On the MODMA dataset under subject-independent five-fold cross-validation, the proposed method outperforms a set of reproduced representative baselines (SVM, EEGNet, InceptionNet, Self-attention-CNN and CNN–LSTM) and achieves 84.8% accuracy with an AUC of 0.899. We further discuss recent contemporary directions (e.g., attention/Transformer-based and emotion-aware expert models) and clarify the scope of our empirical comparisons. Furthermore, experiments comparing different frequency bands and band combinations indicate that joint multi-frequency input can enhance classification performance. This study provides an effective multi-domain fusion approach for the automatic diagnosis of depression based on EEG.

## 1. Introduction

Mental health disorders are one of the leading causes of disability and loss of healthy life expectancy globally, with Major Depressive Disorder (MDD) being a common, highly recurrent, and debilitating emotional disorder that increasingly contributes to the global disease burden [1]. In this context, the study of objective biomarkers based on neurophysiological signals has brought new hope to the diagnosis of mental illnesses. Among various brain imaging techniques, Electroencephalography (EEG), with its millisecond-level temporal resolution, non-invasive nature, relatively low cost, and ease of deployment in clinical settings, is considered an ideal medium for fast screening and auxiliary diagnosis of mental disorders [2]. Research has shown that the brain activity of depression patients, both at rest and during tasks, exhibits significant differences in specific frequency bands, functional connectivity, complexity, and network topological attributes compared to healthy controls [3,4]. These findings provide a solid theoretical foundation for EEG-based objective diagnosis.

Early automation research focused primarily on traditional machine learning methods. These methods typically follow the “feature engineering + classifier” paradigm, where researchers manually extract time-domain, frequency-domain, nonlinear dynamics, or brain network features from EEG signals and input them into classifiers such as Support Vector Machines (SVM) or Random Forests for pattern recognition [5,6]. Although these studies have achieved some success, their performance is severely limited by the quality of feature engineering. The feature selection process is not only labor-intensive but also dependent on expert knowledge, and may fail to capture deep, complex, nonlinear spatiotemporal dynamics in EEG signals, leading to information loss and poor model generalization ability.

In recent years, deep learning techniques have made revolutionary advancements in fields such as computer vision and natural language processing due to their powerful end-to-end feature learning capabilities, and they have rapidly permeated the field of biomedical signal processing [7]. Convolutional neural networks (CNN), recurrent neural networks (RNN), and their variants (such as long short-term memory networks (LSTM) and gated recurrent units (GRU)) have been widely applied to EEG signal analysis, enabling the automatic learning of discriminative feature representations from raw or simply preprocessed data [8,9]. For example, Yan et al. proposed a multi-scale convolutional recurrent neural network (MCRNN), which performed excellently in various psychiatric disorder classification tasks; Saeedi et al. combined CNN with LSTM for depression classification. However, pure temporal models struggle to explicitly model the spatial topology between electrodes [10]. Although CNN-LSTM hybrid models attempt to extract spatial features through convolutional layers, pooling operations may still compromise the integrity of spatial information.

To address the limitations of conventional CNN- and RNN-based EEG models, recent studies have explored more advanced representation learning strategies. Attention- and Transformer-based architectures have been investigated for multi-channel EEG modeling, enabling global dependency modeling in applications such as seizure prediction and emotion recognition [11,12]. Meanwhile, spatial representation strategies, including EEG power-map representations combined with deep learning modules, have been explored for physiological and cognitive state monitoring [13]. In the context of depression assessment, semantic-information-assisted and expert-ensemble frameworks have also been investigated to improve prediction performance [14]. In addition, automated machine-learning approaches have been increasingly applied to biomedical signal analysis, clinical prediction, and computer-aided diagnosis tasks [15,16,17,18]. Motivated by these methodological developments, our work focuses on a lightweight temporal model that preserves multi-channel configuration without pooling; the model learns interchannel dependencies implicitly while ingesting a spatial–frequency EEG tensor sequence and performing end-to-end learning.

In summary, existing EEG decoding methods face limitations in fusing information across time, frequency, and spatial domains. Specifically, one challenge is to automatically capture long-term dependencies in both the time-domain and frequency-domain signals. Another challenge is to effectively utilize the spatial distribution information of the electrodes. However, current approaches often struggle to meet both of these requirements simultaneously.

To address these challenges, this paper presents an improved bidirectional LSTM (BiLSTM) model along with a novel data representation for multi-channel EEG signals. The proposed data structure preserves multi-channel configuration without pooling; the model learns inter-channel dependencies implicitly, and incorporates information from multiple frequency bands. This integrated approach enables a more comprehensive analysis of EEG data across multiple domains. The main contributions of this paper are as follows:

- Providing a reproducible preprocessing-to-tensorization pipeline that converts continuous 128-channel resting-state EEG into fixed-length (2-s) epochs and standardizes the model input as a 4-D tensor of shape (B,1,C,T) for subject-independent evaluation.
- An improved bidirectional LSTM (BiLSTM) network structure is designed to capture the long-term dependencies of multi-domain inputs from a temporal perspective. The BiLSTM encodes temporal features from both the forward and backward directions, and the outputs are concatenated to produce a sequence representation rich in contextual information.
- Designing a BiLSTM-plus-vector-routing classifier where the BiLSTM models temporal dependencies over the T time steps using the full C-channel snapshot at each time step, and vector routing produces vector-based class representations for robust depression recognition.

## 2. Related Work

The automated diagnosis of mental disorders based on EEG has evolved from traditional machine learning methods to deep learning and, more recently, to specialized model designs addressing real-world challenges. This section systematically reviews relevant research and defines the positioning and contribution of this work within the field.

### 2.1. Traditional Machine Learning Methods

Before the widespread application of deep learning, research paradigms mainly relied on a combination of handcrafted feature engineering and shallow classification models. The core of this process lies in extracting discriminative features from multi-channel EEG signals that can characterize the pathological state of mental disorders.

In feature engineering, researchers have explored features across multiple domains. The most commonly used are frequency-domain features, such as the power spectral density and spectral entropy of various frequency bands ( δ,θ,α,β,γ ). For instance, Hosseinifard et al. achieved an accuracy of 83.3% in depression classification by calculating the power spectra of four classical frequency bands and their nonlinear features [4]. Given that the brain is a complex nonlinear system, features like sample entropy, fuzzy entropy, and fractal dimension have been used to quantify the complexity of EEG signals, showing promise in classifying schizophrenia and bipolar disorder. Brain network features are based on graph-theoretic analysis methods that treat different brain regions as nodes, constructing functional connectivity matrices by calculating synchronization likelihood, phase-locking values, and other indicators. These network properties (such as clustering coefficient, path length) are then used as features. Hasanzadeh et al. effectively distinguished severe depression using directed brain network features [19].

In terms of classification models, Support Vector Machines (SVM), K-Nearest Neighbors (KNN), Linear Discriminant Analysis (LDA), and Random Forests (RF) have been widely used. For example, Shen et al. achieved over 97% accuracy in anxiety diagnosis using ensemble learning methods combined with power spectrum and functional connectivity features [20].

Despite some success, these methods have significant limitations: First, feature extraction heavily relies on expert knowledge and trial-and-error, making it cumbersome and difficult to generalize across different diseases or datasets. Second, handcrafted features may fail to fully capture deep and complex patterns inherent in EEG signals, limiting the performance of the model.

### 2.2. Deep Learning Methods

Deep learning has overcome the limitations of handcrafted feature engineering by automatically discovering discriminative features from data in an end-to-end manner, and has become the mainstream approach in current research.

#### 2.2.1. Convolutional Neural Networks (CNNs)

CNNs are widely applied due to their outstanding ability to extract image features. To adapt CNNs to EEG time-series data, researchers typically use two strategies: one is to convert EEG data into images, and the other is to apply 1D convolution directly to the time-series data.

Li et al. treated functional connectivity matrices from five frequency bands as different channels and combined them into three-channel color images to input into CNNs for mild depression classification [21]. Shahriman et al. used functional connectivity matrices directly as input for schizophrenia classification [22]. While effective, these methods may lose fine-grained temporal dynamics in the original signal.

Shoeibi et al. used 1D-CNN to process EEG time-series fragments, preserving the temporal structure, but these methods often lack the ability to model the relationship between spatial channels, typically requiring subsequent fully connected layers for implicit learning, which is inefficient [23].

Convolutional neural networks (CNNs) are widely used to learn spatial–frequency patterns in EEG data. For example, Acharya et al. used a 13-layer CNN to detect depression with high accuracy [24], and specialized EEG-specific CNNs (such as EEGNet) have been proposed to capture both temporal and spatial information. Hybrid CNN architectures have also been explored. Wan et al.’s HybridEEGNet employed parallel CNN branches to learn both synchronization and regional EEG features [25]. Similarly, SparNet (a parallel CNN with an attention module) explicitly fuses multi-region spatial features and frequency-domain inputs, demonstrating that combining spatial and spectral information enhances depression discrimination [26]. However, standard CNNs may lose long-term temporal dependencies and often use fixed-scale kernels that cannot capture multi-scale temporal patterns.

#### 2.2.2. Recurrent Neural Networks (RNNs)

Recurrent neural networks (RNNs), especially long short-term memory (LSTM) and gated recurrent unit (GRU) variants, are used to model the temporal dynamics of EEG signals. LSTM networks mitigate the vanishing-gradient problem of classic RNNs and can learn long-range dependencies in EEG time series. For instance, Seal et al. proposed DeprNet, a deep convolution neural network framework for detecting depression using EEG, demonstrating superior depression recognition performance compared with conventional approaches [27]. Several works combine CNN and RNN: Ozal et al. proposed a deep hybrid CNN–LSTM model for depression detection with high accuracy [28], and Fan et al. and Sharma et al. integrated 1D CNNs with LSTM to jointly capture spatial and temporal EEG characteristics [29]. Similarly, Wang et al. fused a 1D-CNN front end with GRU layers and an attention mechanism (1D-CNN-GRU-ATTN) [30]. Multi-scale RNNs have also been developed: Yan et al. designed a multi-scale CNN–GRU (MCRNN) that blends CNN and GRU to exploit spatio-temporal EEG information, yielding multi-disorder classification (including MDD) with moderate accuracy [31]. Beyond depression-focused studies, BiLSTM-based EEG time-series models have also been explored for other psychiatric conditions; for example, a Bi-LSTM-based model was reported for efficient schizophrenia diagnosis using time-series EEG data (REEDCON 2023) [32]. Nonetheless, pure RNNs (LSTM or GRU) lack explicit spatial feature extraction, and even CNN–RNN hybrids may neglect spectral cues.

However, pure RNN-based models also lack explicit modeling of electrode spatial topologies when handling multi-channel EEG data. While CNN-LSTM hybrid models attempt to address this, the local receptive field and pooling operations in CNNs may disrupt the completeness of spatial information.

#### 2.2.3. Multi-Domain Fusion

Many studies emphasize feature extraction in specific domains. Time-domain approaches often use raw EEG or temporal statistics, whereas frequency-domain methods compute band powers or entropy. For example, Li et al. found that band-power features in alpha/beta bands were highly discriminative for MDD [33]. Spatial-domain information is captured via functional connectivity or channel topographies. Graph-based methods construct brain networks: Liu et al. built EEG graph adjacency matrices from channel correlations and used graph CNNs for classification [34]. However, most existing works focus on single-domain features. Ay et al. demonstrated a CNN–LSTM model per hemisphere (time-spatial fusion) for MDD with 85% accuracy, but did not incorporate frequency features [28]. Likewise, Saeedi et al. fused LSTM with 1D- and 2D-CNN on EEG connectivity for MDD recognition; while effective in capturing time and spatial patterns, this method still omitted explicit frequency-domain signals. TSF-MDD explicitly addresses this gap by constructing a 4D temporal–spatial–frequency representation of EEG and processing it with 3D-CNN [35]. Such multi-domain fusion approaches report superior performance: for instance, combining all frequency bands generally outperforms single-band or full-band inputs.

In summary, CNN-based models excel at learning spatial (or spatial–frequency) EEG features, whereas RNN-based models capture temporal dynamics. CNN–RNN hybrids attempt to harness both, but in practice simple stacking often underutilizes one domain. Prior evaluations show that 1D-CNN and 1D-CNN–LSTM baselines (which rely mainly on temporal features) lag behind models that integrate spatial and frequency. EEGNet and other shallow CNNs (temporal-only) similarly fail to leverage inter-channel structure. On the other hand, models focusing on connectivity (graph or attention networks) capture spatial relationships but may overlook raw time–frequency content. Pure RNNs (LSTM/GRU) trained on EEG sequence features can detect temporal patterns, but require careful design to avoid gradient issues, and they do not inherently exploit spatial layout. Consequently, hybrid models such as CNN–LSTM, CNN–GRU, or 3D-CNN have become common: for example, Salama et al. used 3D-CNNs on volumetric EEG inputs to jointly model temporal and spatial variations [36], and Zhang et al. combined attention-based GCNs with LSTMs [37]. These architectures achieve high classification rates, but all reflect a tradeoff: either lose detailed domain information or require elaborate data representations (e.g., connectivity matrices).

Despite the high accuracies reported, existing methods have noteworthy limitations. Many algorithms are evaluated under subject-dependent splits, inflating performance by allowing test samples from the same subjects as training. More critically, most studies do not fully exploit the multi-band, multi-channel nature of EEG. As noted in recent reviews, EEG signals exhibit rich temporal, spatial, and spectral patterns, yet many current studies focus exclusively on temporal, frequency, or spatial features [38,39]. Similarly, other analyses conclude that inadequate multi-domain fusion is a common problem in deep EEG models. Some methods incorporate functional connectivity to capture inter-channel relations, but this may still “ignore valuable time-frequency features in the raw signals”. Moreover, conventional CNN filters (even separable ones) use fixed scales and pooling that can lose temporal detail, while simple CNN+LSTM stacks may fail to effectively blend spatial and temporal representations. Notably, recent deep MDD studies seldom encode electrode topology and spectral bands jointly into the input. For example, although SparNet and TSF-MDD integrate spatial and frequency cues through clever architectures, they rely on CNN backbones and do not leverage sequential models. The need remains for models that can natively ingest EEG tensors preserving both spatial layout and multi-band content, and then apply powerful sequence learning.

In light of these gaps, we propose an improved BiLSTM architecture with a spatial–frequency EEG tensor input. By organizing multi-channel EEG across frequency bands into a higher-dimensional representation, our model explicitly maintains inter-channel topography and spectral structure. The bidirectional LSTM layers can then capture temporal dependencies from both forward and backward directions, addressing the gradient limitations of uni-directional RNNs. This design directly targets the limitations of prior work: it enables end-to-end learning of spatio-spectral features over time, whereas previous CNN-only or hybrid models often neglected one of these domains. In summary, our approach aims to fully exploit multi-domain EEG information within a deep sequence model, addressing the deficiencies of existing EEG-depression classification frameworks.

#### 2.2.4. Transformer-Based EEG Architectures

Beyond CNN/RNN hybrids, recent EEG modeling increasingly adopts attention/Transformer paradigms to capture global inter-channel interactions and long-range dependencies, showing strong performance in tasks such as seizure forecasting and EEG-based affect recognition. Another emerging direction for depression assessment is to incorporate emotion-related cues and route samples to specialized experts, which aims to mitigate distribution shifts across affective contexts. Meanwhile, the biomedical ML community emphasizes interpretability and automated pipeline design to improve transparency and facilitate deployment.

The qualitative comparison table is shown in Table 1. This table provides an overview of recent approaches and their respective tasks, input representations, and key ideas. In comparison to these works, recent studies have demonstrated the effectiveness of attention mechanisms, topographic representations, and deep sequence models for EEG-based analysis across various mental and neurological tasks. Transformer-based approaches, such as B2-ViT, highlight the importance of global channel interaction modeling through attention, while our method preserves multi-channel configuration without pooling; the model learns inter-channel dependencies implicitly, and employs BiLSTM combined with vector-based routing.Efficient Transformer encoders used in emotion recognition further motivate the adoption of attention-based modeling; however, our work specifically targets depression detection and explicitly models EEG signals as spatial–frequency tensor sequences. Prior fatigue detection studies leveraging 2D EEG power maps with recurrent and attention modules support the value of topographic representations, which we extend by stacking multi-band frames and applying bidirectional temporal modeling. Emotion-aware ensemble frameworks illustrate the benefit of semantic routing across experts, whereas our approach instead emphasizes multi-domain EEG fusion within a single-dataset setting to reduce dependency on auxiliary annotations. Comparative studies evaluating conventional deep learning baselines, such as CNN-LSTM and BiLSTM architectures, provide useful context for performance benchmarking, while our contribution lies in reconstructing spatial–frequency EEG frames and introducing vector-based routing to enhance spatial feature preservation. Finally, feature-based machine learning methods using wavelet transforms complement the existing literature, in contrast to our fully end-to-end deep sequence learning framework that jointly captures spatial, spectral, and temporal dependencies.

## 3. Method

**Figure 1:**
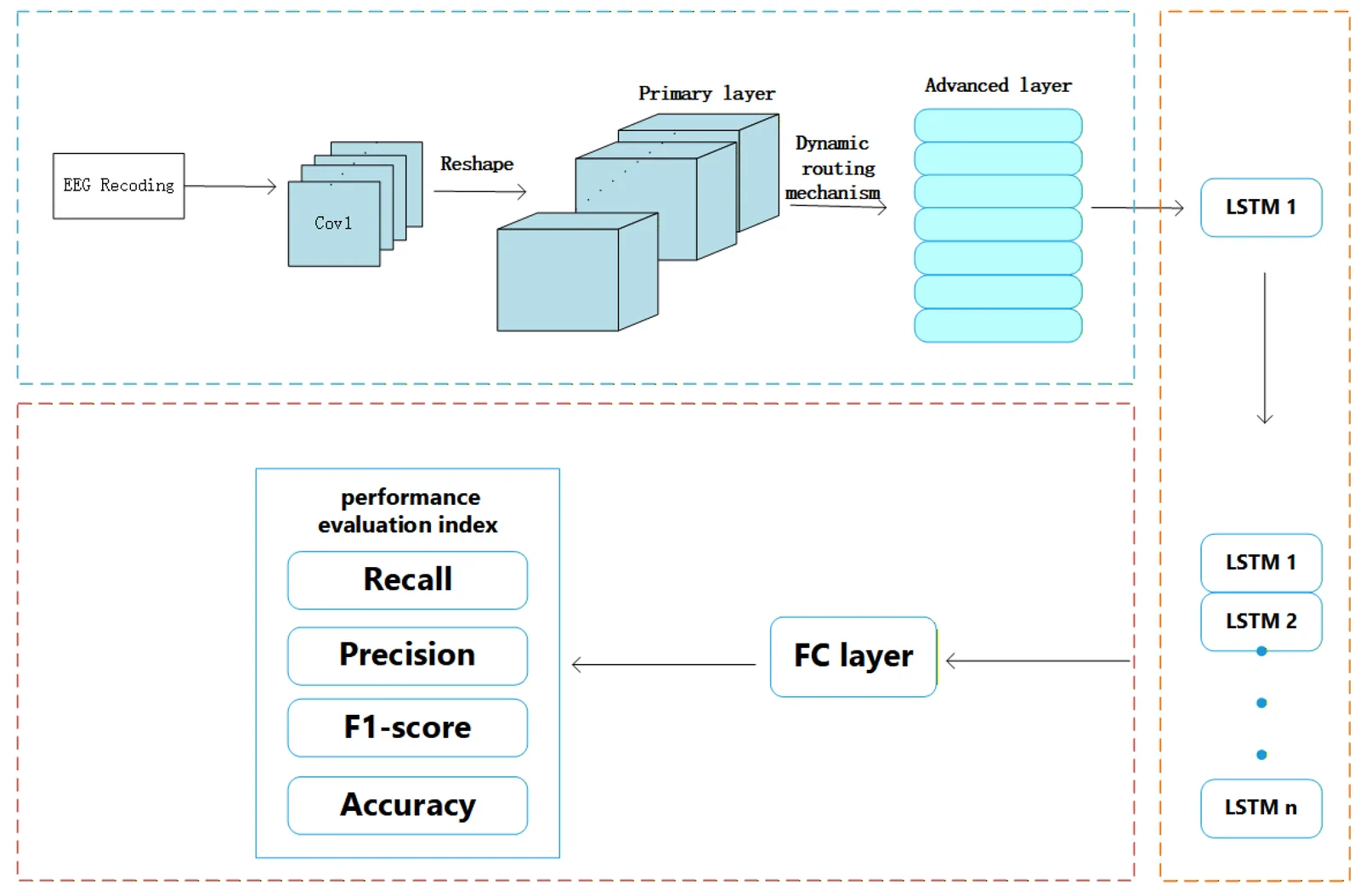
Depression diagnosis framework.

This research introduces a bidirectional LSTM (BiLSTM) model for EEG-based depression diagnosis. The depression diagnosis framework is shown in Figure 1. The innovation comes in improving the traditional LSTM model to handle the complex temporal and spectral correlations present in EEG signals. This is vital for correct categorization of mental disorders such as depression.

### 3.1. Data Preprocessing

The model’s success depends on high-quality input data. This study applies a systematic preprocessing pipeline to turn raw, noisy multi-channel EEG signals into organized three-dimensional tensors rich in temporal, spatial, and frequency data.

#### 3.1.1. Artifact Removal

The dataset used in the experiment is MODMA, which includes EEG data from 23 MDD patients and 29 healthy control (HC) subjects with 128 EEG channels [40]. Electrode positions follow the international 10–20 naming convention (extended with intermediate locations to support high-density coverage), providing a near-uniform sampling of scalp potentials over anterior–posterior and left–right directions. Following the conventional interpretation of 10–20 labels, electrodes with similar prefixes are associated with nearby cortical areas: prefrontal/frontal (Fp/AF/F), central sensorimotor (FC/C/CP), parietal (P), occipital (PO/O), and temporal (FT/T/TP) regions. Midline electrodes (z) primarily sample medial cortices along the sagittal plane. The subjects were recruited from inpatient and outpatient patients at the Second Hospital of Lanzhou University, diagnosed with MDD according to the DSM-IV criteria. The MDD patients include 16 males and 7 females, aged 16–56 years, while the healthy controls consist of 20 males and 9 females, aged 18–55 years. After excluding subjects with incomplete EEG data, 23 MDD patients and 29 healthy controls’ resting-state EEG data were used for the experiment. The EEG data from each subject was preprocessed as described earlier, filtered and artifact-free, and divided into 2-s non-overlapping sliding windows. After data normalization and structure reconstruction, the final dataset contains 21,878 samples (HC:MDD = 11,722:10,156). Given the small imbalance ratio, no explicit class re-weighting or resampling techniques were applied. Instead, accuracy was employed as the primary evaluation metric to ensure robustness against minor class imbalance.

Raw EEG signals are typically contaminated by various physiological artifacts (such as eye movement and muscle activity) and non-physiological artifacts (such as power line interference). The preprocessing pipeline was completed using the MNE-Python 1.9.0 with the following steps:

1. A band-pass filter with a 0.5–70 Hz finite impulse response was applied to preserve the valid EEG activity frequency range, while removing very low-frequency drifts and high-frequency noise. As a result, frequency components above 70 Hz were excluded from subsequent analysis, and the gamma band in this study corresponds to the 30–70 Hz range.
2. A notch filter with a 50 Hz frequency (based on the local power line frequency) was used to eliminate electrical grid interference.
3. Data segments containing large motion artifacts were identified and removed using visual inspection and an automatic algorithm (e.g., thresholding with an amplitude exceeding ±100 μV). Persistent electrode channels with poor signal quality were interpolated or excluded.
4. The original reference was converted to an average reference across the entire brain to minimize the impact of the reference electrode location and provide a more global view of brain activity.
5. The ICA algorithm was applied to decompose the multi-channel signals into statistically independent components. By analyzing the spatiotemporal features of each component, artifacts related to eye movements, blinking, and muscle activity were identified and removed using manual or automatic tools such as ICLabel.
6. The continuous resting-state data was segmented into 2-s non-overlapping windows to increase the sample size. Finally, Z-score normalization was conducted in a fold-wise manner to prevent information leakage: for each GroupKFold split, the mean and standard deviation were estimated exclusively from the training-fold epochs and then consistently applied to normalize the corresponding training, validation, and test data within the same fold.
7. To further eliminate potential data leakage, EEG signals were segmented using fixed-length, non-overlapping windows. Since no temporal overlap exists between adjacent segments, no shared samples or temporal redundancy are introduced across epochs. Moreover, window segmentation was performed independently for each subject prior to cross-validation, ensuring that no temporally adjacent data from the same subject were split across training and test folds.

Several preprocessing steps above are included primarily to prevent non-neural confounds and information leakage under subject-independent evaluation. Removing them can lead to clinically invalid comparisons (e.g., models exploiting ocular/muscle artifacts or leakage-related redundancy rather than depression-related neurophysiology). Therefore, in this work we focus on robustness analyses over preprocessing degrees of freedom that remain meaningful under GroupKFold while keeping leakage-prevention and artifact-control steps fixed.

#### 3.1.2. Feature Representation and Leakage Control

Unlike traditional methods that rely on handcrafted spectral features, the proposed model directly operates on minimally preprocessed EEG signals. After band-pass filtering and resampling, the raw EEG epochs are input into the network, allowing the BiLSTM and vector-representation layer to automatically learn discriminative temporal and frequency-sensitive representations in an end-to-end manner. Potential spectral leakage was minimized through a combination of preprocessing and model design choices. First, a finite impulse response band-pass filter was applied prior to epoch segmentation, effectively suppressing out-of-band frequency components. Second, fixed-length, non-overlapping windows were employed, which prevents artificial correlations typically introduced by overlapping windows. Furthermore, instead of relying on explicit Fourier-based spectral decomposition, which is inherently sensitive to windowing effects, the model learns frequency-related patterns implicitly through temporal modeling, further reducing the risk of spectral leakage caused by the windowing process. The window length was chosen to be 2 s, as it provides a balance between temporal resolution and signal stationarity. Shorter windows may fail to capture enough temporal context for modeling EEG dynamics, while longer windows introduce greater non-stationarity and increase computational complexity. The sensitivity analysis reported in Section 4.2.2 empirically validates this design choice, demonstrating that the 2-s window yields superior and more stable performance compared with alternative window lengths.

### 3.2. Multi-Domain Data Structure Reconstruction

After loading each EGI recording, we retain EEG channels only and perform a preprocessing pipeline consistent with our released codebase. Specifically, the signal is resampled to 150 Hz to standardize the temporal resolution across subjects. We segment the continuous recording into non-overlapping 2-s epochs, yielding an epoch-wise data tensor $X\in R^{C\times T}$ , where C = 128 denotes channels and T is the number of samples per 2-s window after resampling. To guarantee a consistent channel dimension, recordings with more than 128 EEG channels are truncated to the first 128 channels, whereas recordings with fewer channels are zero-padded to 128 channels.

Normalization (Z-score) is performed fold-wise to avoid leakage. Under participant-independent 5-fold GroupKFold splitting, we compute the mean μ and standard deviation σ only from the training fold epochs (i.e., over the epoch dimension, at every (c,t) location), clamp very small σ with a floor ( $1\times e^{-6}$ ) for numerical stability, and then apply the same (μ,σ) to normalize the training fold data as well as the corresponding validation/test data in that fold (in-place for memory efficiency). This protocol matches the participant-grouped evaluation design (no subject overlap across train/test within a fold).

For model compatibility, each epoch matrix is expanded to a 4-D tensor (B,1,C,T) by adding a singleton dimension. Inside the network, the input is reshaped to (B,T,C) via squeeze-and-permute so that the BiLSTM processes a length-T temporal sequence while observing the full C-channel snapshot at each time step; the resulting sequence features are then passed to a dynamic-routing vector-representation classifier (i.e., routing-based vector units) for decision making. More importantly, our BiLSTM processes the full C-channel snapshot at each time step (B,T,C) and the routing layer aggregates vectorized temporal features; therefore preserving a dense, non-interpolated channel dimension is a better match to the model’s inductive bias.

Finally, in addition to the default full-band implementation used for the main pipeline, we also conducted frequency-band ablation/evaluation by re-running the same preprocessing/training procedure while changing the band-pass range to the canonical δ/θ/α/β/γ bands, and further testing representative band combinations. Additionally, the method for achieving band combinations (allbands) is: for a given epoch $X\in R^{C\times T}\left(C=128\right)$ , we first obtain five sub-band epochs $X^{\delta },X^{\theta },X^{\alpha },X^{\beta },X^{\gamma }$ by applying the corresponding band-pass filters. We then construct the combined-band input by concatenating these sub-band signals along the channel dimension: $X^{all}=concat\left(\left[X^{\delta },X^{\theta },X^{\alpha },X^{\beta },X^{\gamma }\right],dim=channel\right)\in R^{(5C)\times T}$ . Accordingly, the network input tensor becomes (B,1,5C,T) for allbands, while it remains (B,1,C,T) for fullband or any single sub-band.

EEG signals are typically considered for different frequency bands. Based on the frequency, EEG rhythmic activity can be divided into five basic frequency bands: δ band (0.5–4 Hz), θ band (4–8 Hz), α band (8–13 Hz), β band (13–30 Hz), and γ band (30–70 Hz). The δ band is recorded in infants, adult fatigue, or sleep states. The θ band is generally associated with situational memory and cognitive memory [41]. The α band is a basic rhythm in EEG, reflecting brain attention processing [42,43], and it is found to be significantly reduced in bipolar disorder patients [44]. The β band reflects emotional and cognitive processing [44], and studies have found that there is a positive correlation between the amplitude of β band activity in the left frontal lobe and positive emotions in depression patients [45]. The γ band is the highest frequency band and becomes more prominent during attention concentration. Abnormal γ band activity has been observed in schizophrenia patients, which is considered to underlie confusion and cognitive defects [10]. In summary, analyzing the EEG frequency domain features of mental disorder patients based on these basic rhythms is effective [46]. These single-band and multi-band results quantify how different spectral components contribute complementary information to depression recognition beyond the default band-limited setting.

### 3.3. Improved BiLSTM Network Architecture

**Figure 2:**
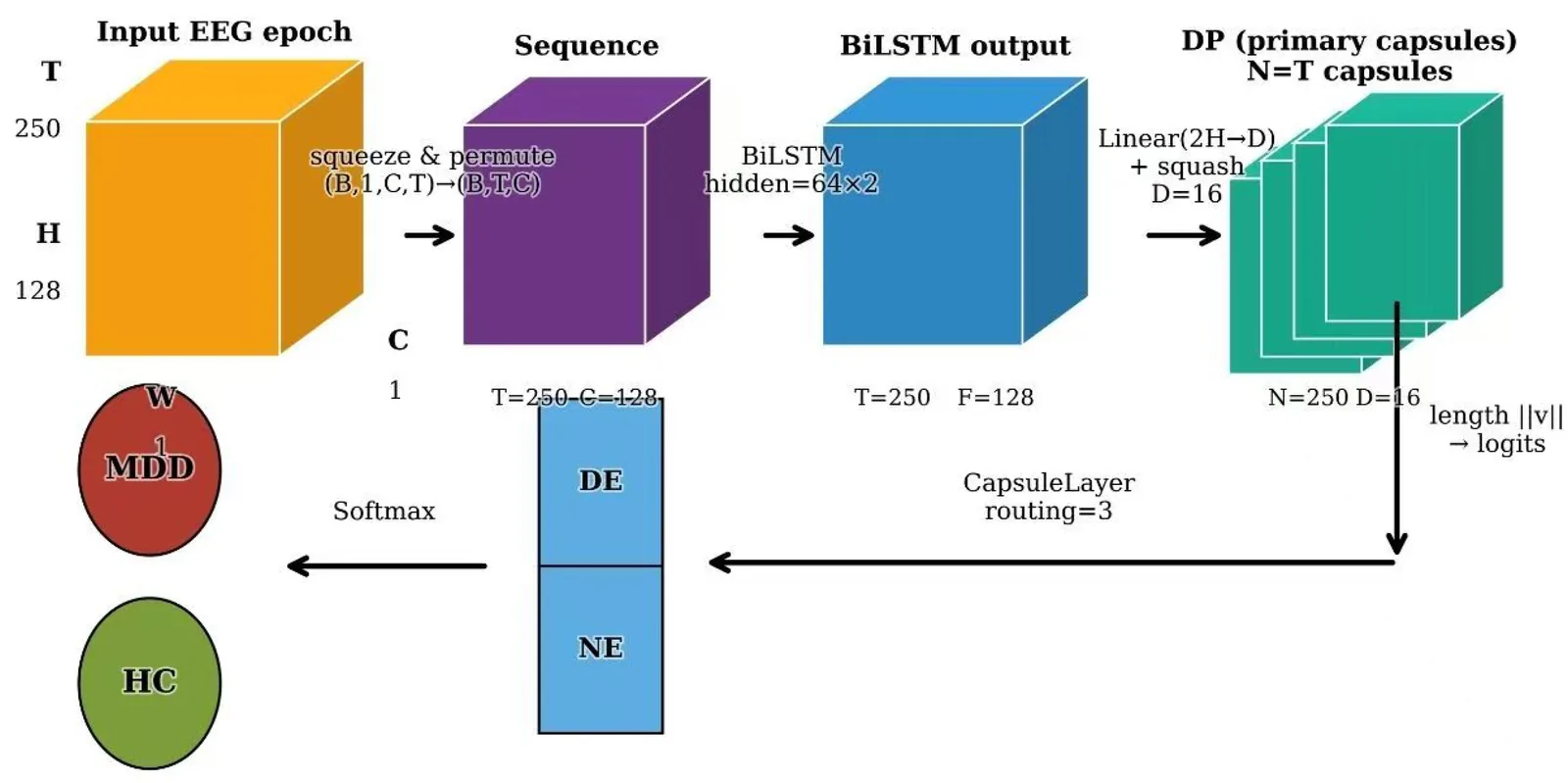
Classification model structure based on the Improved LSTM.

The Improved BiLSTM model proposed in this paper is an end-to-end deep learning architecture designed to extract temporal dependencies while preserving multi-channel configuration without pooling; inter-channel dependencies are learned implicitly from the C-channel snapshot at each time step. Its overall structure is shown in Figure 2, comprising the following components:

#### 3.3.1. Bidirectional Long Short-Term Memory Network (Bi-LSTM)

Bi-LSTM is responsible for extracting rich temporal context features from the reconstructed data sequence and parsing the input three-dimensional data frame sequence. The BiLSTM simultaneously exploits past and future context, which is advantageous on long, noisy sequences (e.g., EEG) compared with a unidirectional RNN/GRU.Specifically, each spatial frame in the sequence is flattened into a vector and input to Bi-LSTM in temporal order. The forward and backward LSTM units capture long-term dependencies from both the forward and backward perspectives using their gating mechanisms (input gate, forget gate, and output gate). For a given time step t, the forward hidden state $h_{t}^{f}$ and backward hidden state $h_{t}^{b}$ are concatenated to form a comprehensive context-aware representation:

(1) $$h_{t}=\left[h_{t}^{f};h_{t}^{b}\right]\in R^{2H}$$

This process enables the model to understand the temporal dynamics of EEG patterns, outputting a feature sequence $\left\{h_{1},h_{2},\ldots ,h_{T}\right\}$ rich in temporal information.

#### 3.3.2. Vector-Representation Layer with Dynamic Routing Concept

Instead of the conventional convolution–pooling path that represents spatial features by scalar activations followed by down-sampling, this branch uses vectorized feature units whose direction encodes high-level attributes (e.g., pose/arrangement) while the magnitude encodes the detection confidence of that attribute. To avoid disrupting the multi-channel configuration normally caused by pooling, dynamic routing adaptively assigns predictions from lower layers to higher-level units, enabling the model to learn inter-channel dependencies implicitly.

Let the outputs from the lower layer be organized into a matrix $U=\left[u_{1},\ldots ,u_{n_{1}}\right]\in R^{d\times n_{1}}$ . For the j-th higher-level vector unit, we introduce a corresponding learnable transformation $W_{j}\in R^{d^{\prime }\times d}$ , yielding the prediction matrix:

(2) $${\hat{U}}_{j}=W_{j}U$$

where the i-th column of $\hat{U}j$ , denoted $\hat{u}ij$ , is the predicted output from the i-th lower-level vector unit to the j-th higher-level vector unit. Let $a_{j}=softmax\left(b_{j}\right)\in R^{n_{1}}$ be the normalized coupling weights that control how information from the $n_{1}$ lower-level vector units is routed to the j-th higher-level vector unit. The pre-activation for unit j is then given by:

(3) $$s_{j}={\hat{U}}_{j}a_{j}$$

The softmax normalization is performed along the j-th dimension to ensure that $\sum_{i}\alpha_{ij}=1$ . A squash nonlinearity normalizes the output magnitude to [0, 1):

(4) $$v_{j}=\frac{s_{j}}{\left|\right|s_{j}\left|\right|}\frac{\left|\right|s_{j}\left|\right|}{1+\left|\right|s_{j}\left|\right|}$$

The update rule for the coupling coefficients can be written in matrix form as:

(5) $$b_{j}\leftarrow b_{j}+{\hat{U}}_{j}^{T}v_{j}$$

(6) $$a_{j}=softmax\left(b_{j}\right)\in R^{n_{1}}$$

and the routing procedure, which includes softmax normalization, weighted summation, squashing, and updating, is iterated a few times until convergence.

#### 3.3.3. Margin Loss

Because our model outputs a vector for each class with its Euclidean length encoding the confidence in that class prediction, we adopt a margin loss as the training objective in lieu of a standard cross-entropy criterion. This margin-based loss inherently encourages better class separation: it forces the true class’s output vector norm to exceed an upper threshold $m^{+}$ while constraining non-target class norms below a lower threshold $m^{-}$ . Such enforcement creates a clear margin between classes in the output space, yielding more discriminative representations of depressive vs. healthy EEG patterns. Moreover, margin loss addresses imbalanced confidence levels between classes by down-weighting the loss contribution of absent classes using the factor λ . Since each class corresponds to a vector output whose Euclidean norm encodes the model’s confidence, we adopt a margin loss as the training objective. Let $T_{k}\in \left\{0,1\right\}$ be the indicator for class *j*. The loss is defined as

(7) $$L_{margin}=\sum_{k}T_{k}*max(0,m^{+}-\left|\right|v_{k}{\left||\right)}^{2}+\lambda \left(1-T_{k}\right)*max(0,||v_{k}\left|\right|-m^{-}{)}^{2}$$

where $T_{k}$ is 1 when class k is present, $m^{+}$ and $m^{-}$ are upper and lower margins, and λ is a weight coefficient. This alignment scheme renders margin loss a more superior choice in our architecture. It takes full advantage of the model’s vectorized confidence encoding to enforce robust inter-class distinctions and enhance the discrimination of learned EEG features.

#### 3.3.4. Model Construction and End-to-End Inference

Each sample is organized as (B,1,C,T) : batch size *B*, spatial-mapping channels *C*, and time steps *T*. Before temporal encoding we reshape to (B,T,C) to feed the sequence along its temporal dimension, consistent with the original specification.

1. Bidirectional LSTM temporal encoding.This produces features of shape (B,T,2·hidden) . No extra batch normalization or activation is applied immediately afterward, as in the original design.
2. Primary fully connected (vectorization). A linear map converts (B,T,2·hidden) to $\left(B,T,dim_{v}ec\right)$ , yielding vectorized representations for each time step; the squash nonlinearity in (4) constrains their magnitudes to [0, 1]. This stage places the temporal features into a vector space amenable to the subsequent routing mechanism.
3. Vector routing layer (dynamic routing). Applying (2) to obtain ${\hat{U}}^{j|i}$ , (3) for coupling, and (4) for squashing, with (5) to update $b_{ij}$ , we iterate until convergence and output a set of higher-level vectors $v_{j}$ .
4. Classification readout. For each $v_{j}$ , the magnitude $\left|\right|v_{j}\left|\right|\in \left[0,1\right]$ serves as the activation confidence. Multiplying by a learnable scaling factor yields the logits; training uses margin loss, whereas inference selects the class with the maximum confidence.

Through this systematic design, the Improved BiLSTM Network model aims to achieve deep, efficient, and robust feature learning and classification for depression EEG signals.

## 4. Results

We prepared and carried out a number of rigorous tests to thoroughly assess the performance of the suggested Improved BiLSTM Network model in the depression diagnosis job, particularly in data-limited settings. This part describes the experimental setup, dataset specifics, assessment measures, and methodically confirms the model’s efficacy through ablation trials, comparisons with baseline models.

### 4.1. Experimental Setup

#### 4.1.1. Dataset and Preprocessing

This study used the publicly available MODMA dataset for the core experiments. The data were collected from 128 electrode channels, with a sampling frequency of 250 Hz. We strictly followed the preprocessing steps outlined in Section 3.1, including 0.5–70 Hz bandpass filtering, 50 Hz notch filtering, ICA artifact removal, full-brain average re-referencing, and Z-score normalization. The model was trained using the AdamW optimizer with a learning rate of $2\times e^{-3}$ , and early stopping was employed to prevent overfitting. The batch size for model training is set to 64.

#### 4.1.2. Evaluation Protocol and Baseline Models

This study adopted a five-fold cross-validation strategy based on participant grouping to ensure the independence of participants during the evaluation process. Specifically, we used the GroupKFold method to divide the data according to the participants, resulting in five folds. Approximately 80% of the participant data in each fold was used for training, and the remaining 20% was used for testing, ensuring that there were no overlapping participants between the training set and the test set in the same fold. That is to say, in each fold, the model always tested on new participant data that it had not seen during the training process. For each fold’s training set, we further divided 20% of the data into an internal validation set, which was used for model hyperparameter tuning and training early stopping. All data preprocessing steps (including Z-score normalization) were strictly based on the training set of each fold: we only calculated the mean and standard deviation required for normalization using the training set and applied these normalization parameters to the validation set and test set of that fold. This process was repeated five times, allowing each participant’s data to be included in the test set exactly once. This participant-independent cross-validation scheme can more reliably evaluate the model’s generalization ability to new participant data. Representative methods from current research were selected as comparison models. It should be noted that many existing studies use within-subject validation or private datasets, making it difficult to directly compare results. Therefore, this study reproduced the following classical models for a fair comparison:

- SVM (Support Vector Machine) [47], as a representative of traditional machine learning, uses handcrafted power spectral density features as input and performs classification decisions via a kernel function.
- InceptionNet [48], InceptionNet is a deep convolutional neural network (CNN) architecture that introduces the idea of inception modules to efficiently utilize multiple convolutional filters with different kernel sizes, thereby capturing information at various levels of granularity within the same layer.
- EEGNet [49,50], a lightweight architecture based on depthwise separable convolutions, is specifically designed for EEG signal processing and includes two key modules: temporal convolution and spatial convolution, performing excellently in several brain-computer interface benchmark tests.
- CNN-LSTM [39], which combines Convolutional Neural Networks and Long Short-Term Memory Networks, first extracts spatial features using 1D-CNN and then inputs the features into the LSTM layer to capture temporal dynamics, aiming to model both the spatial and temporal dimensions of EEG signals.
- Self-attention-CNN [51], which combines Convolutional Neural Networks (CNN) and self-attention mechanisms, starts with convolutional layers to extract spatial features from input data and then uses self-attention to capture long-range dependencies throughout the feature map. Self-attention-CNN combines the characteristics of CNN for local feature extraction and self-attention for global context to successfully capture both local and long-range dependencies in time-series or spatial data, making it especially ideal for applications like EEG signal analysis.

It encodes long-term temporal dependencies through the Bidirectional LSTM layer, generates primary vector units, and then explicitly models the feature space hierarchy using the iterative routing mechanism. This architecture avoids information loss from traditional pooling and preserves multi-channel configuration; inter-channel dependencies are learned implicitly via vector units and routing.

Evaluation was conducted using independent 5-fold cross-validation to ensure the robustness and generalization ability of the results. Performance evaluation metrics include Accuracy, Precision, Recall, F1-Score, and Area Under the Curve (AUC).

Subject-independent cross-validation was strictly enforced using a group-wise splitting strategy. Specifically, a 5-fold GroupKFold protocol was adopted, where subject identifiers were used as grouping labels, ensuring that all EEG epochs from the same subject appeared exclusively in either the training or test set of a given fold. This design completely prevents subject-level information leakage and avoids overly optimistic performance estimation.

### 4.2. Results and Analysis

#### 4.2.1. Overall Performance Comparison

As shown in Table 2, the Improved BiLSTM Network model consistently outperforms the reproduced representative baselines on MODMA under subject-independent five-fold cross-validation. Specifically, Improved BiLSTM Network achieved an accuracy of 84.8% and an AUC value of 0.899, consistently outperforming the reproduced baselines. SVM, as a representative of traditional machine learning methods, performed relatively poorly, highlighting the limitations of handcrafted features in capturing complex spatiotemporal dynamic patterns. Although EEGNet, as a lightweight deep learning model, performed reasonably well, its fixed convolution kernels and pooling operations may limit its ability to capture multi-scale spatiotemporal features. InceptionNet utilizes parallel multi-scale convolutional kernels and spatial attention mechanisms. Its actual performance is comparable to EEGNet, DeprNet, and CNN-LSTM, indicating that this method did not demonstrate a significant advantage under the subject-independent conditions employed in this study. The performance of the CNN-LSTM model was similar to that of EEGNet, suggesting that the simple stacking of CNN and LSTM did not effectively achieve deep integration of spatiotemporal features, and the LSTM model may suffer from the vanishing gradient problem. Improved BiLSTM attained 84.7% accuracy versus 82.9% for Self-attention-CNN, establishing it as the top-performing model. It also achieved a slightly higher precision and recall than the Self-attention-CNN. The higher recall means that the Improved BiLSTM identifies a greater proportion of true positive events, such as it misses fewer relevant EEG occurrences, which is crucial in applications where failing to detect an event (false negatives) is particularly undesirable. At the same time, its strong precision indicates that most of its positive predictions are correct, such as, low false-positive rate, so the model is not simply over-flagging events to boost recall. The F1-score of the Improved BiLSTM is essentially on par with that of the Self-attention-CNN, reflecting that the BiLSTM’s gains in recall did not come at the expense of precision. Instead, both precision and recall are high, suggesting a well-balanced classification performance.

**Figure 3:**
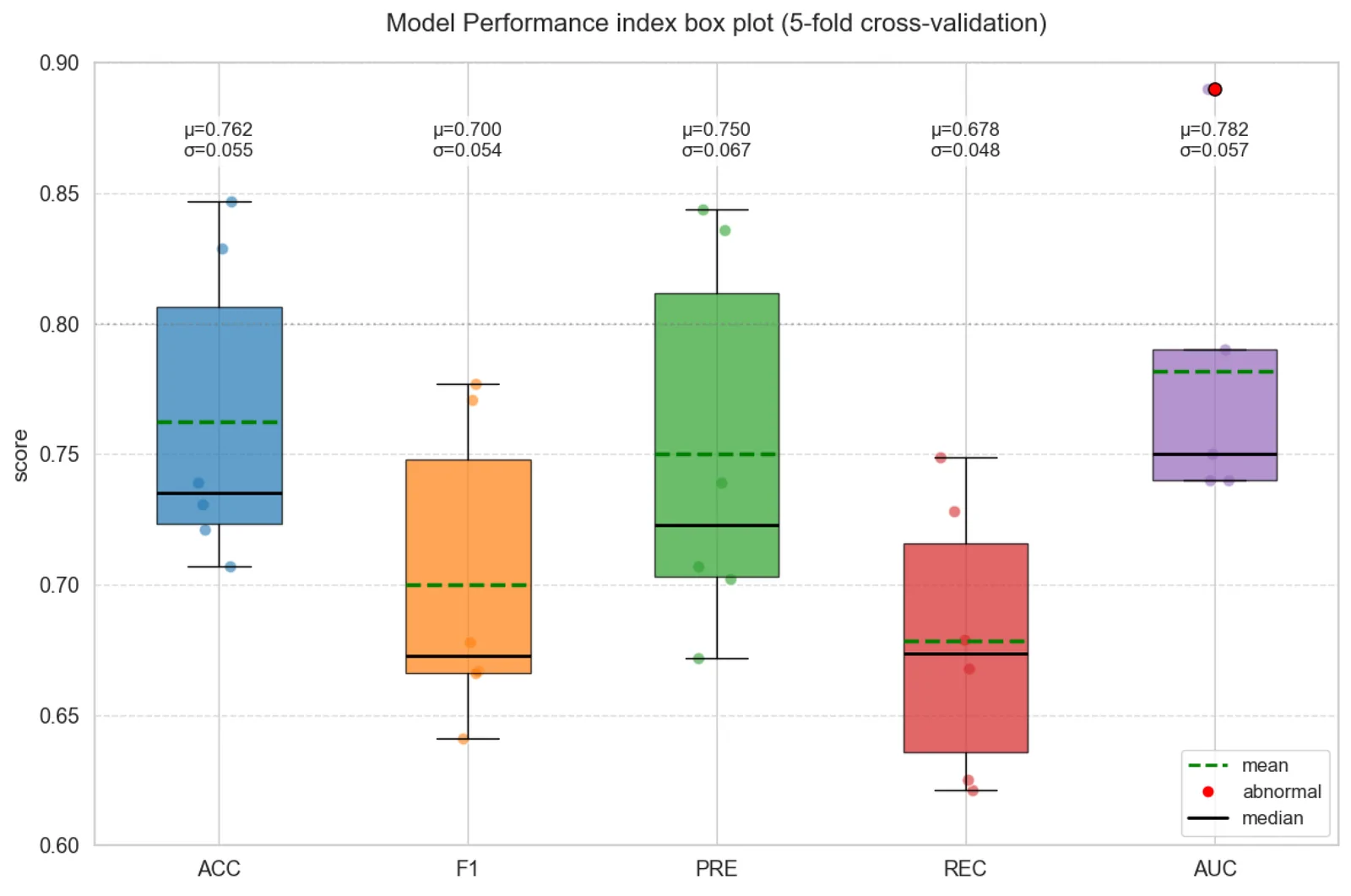
Model performance metric boxplot.

The performance metrics of the models under 5-fold cross-validation are shown in the boxplot in Figure 3. The fold-wise boxplot (Figure 3) indicates a relatively compact inter-fold dispersion under subject-independent GroupKFold, which is inconsistent with severe overfitting behavior typically characterized by high variance across folds on small cohorts.

#### 4.2.2. Hyperparameter Sensitivity Analysis

To empirically justify the selection of key hyperparameters, a sensitivity analysis was conducted on several critical configurations of the proposed Improved BiLSTM Network. Specifically, the BiLSTM hidden size, vector dimension, dynamic routing iterations, and EEG window length were varied independently, while keeping all other parameters fixed. Table 3 clearly indicate that the proposed model exhibits stable performance within a reasonable range of hyperparameter values. When the hidden size of the BiLSTM is increased beyond 64, only marginal performance gains can be achieved, yet this comes at the expense of significantly higher computational complexity. Likewise, when the Vector-Representation Layer exceed 16 and the routing iterations go beyond 3, there is no substantial improvement in performance. Thus, the selected configuration represents a well-balanced trade-off among performance, robustness, and computational efficiency.

#### 4.2.3. ROC Curve Analysis

**Figure 4:**
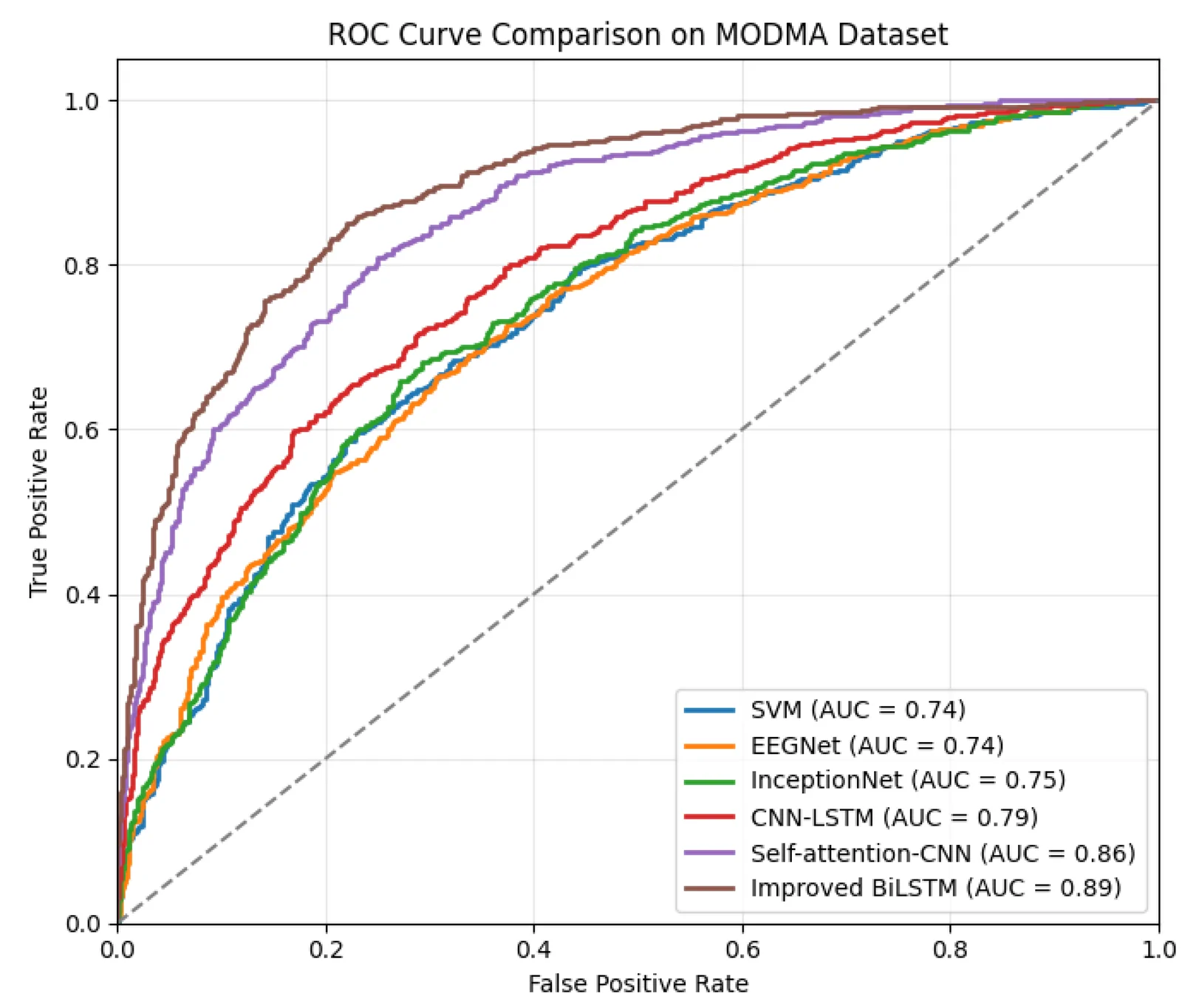
ROC curve comparison.

**Figure 5:**
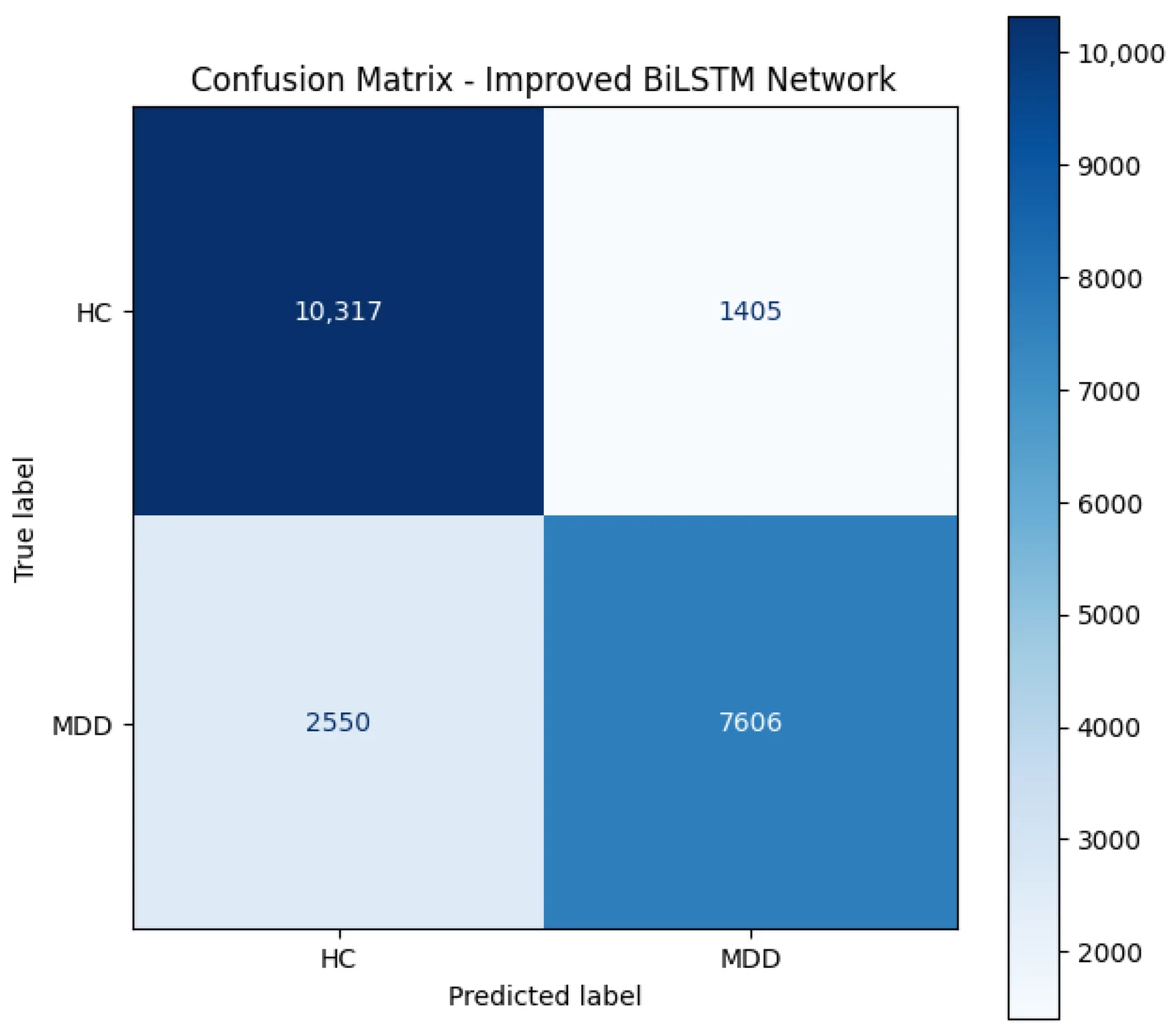
Confusion matrix.

Figure 4 presents the Receiver Operating Characteristic (ROC) curves of six different models on the MODMA dataset, alongside their Area Under the Curve (AUC) values. In Figure 5, the Improved BiLSTM Network clearly yields the best ROC performance among the compared models. Its curve is consistently closer to the top-left (higher TPR for any given FPR) than the others, corresponding to the highest AUC of 0.89. In contrast, the traditional SVM and the compact EEGNet baseline both produce AUCs of 0.74, with ROC curves that hug closer to the diagonal chance line—indicating relatively weaker discrimination. The InceptionNet model performs only marginally better (AUC 0.75), suggesting limited improvement in capturing EEG patterns over EEGNet. The CNN-LSTM baseline shows a moderate enhancement (AUC 0.79), while the Self-Attention CNN achieves a notably higher 0.86 AUC. These differences are reflected in the ROC curves’ shapes: for example, the Self-Attention CNN’s curve lies above the CNN-LSTM’s curve across most of the range, and the Improved BiLSTM’s curve in turn stays above all others. This progression of ROC curves and AUC values demonstrates the growing efficacy of incorporating advanced temporal-spatial feature fusion—culminating in the proposed Improved BiLSTM’s superior performance.

Notably, when compared to other models, the ROC curve of the Improved BiLSTM maintains a higher True Positive Rate at nearly every False Positive Rate. This highlights its enhanced ability to recognize the positive class while effectively keeping false alarms at a low level. In practical applications, across a broad spectrum of decision thresholds, the Improved BiLSTM can identify a larger proportion of depressed subjects for the same false positive rate, which reflects its robust and stable classification capability. Its Area Under the Curve advantage (0.89 compared to 0.86 of the next-best model, and significantly higher than the approximately 0.74–0.79 of the others) indicates a more reliable discriminative performance that is independent of a specific threshold. Such a high AUC implies that the model’s predictions remain effective even when the decision boundary changes, which is crucial for real-world scenarios where the optimal threshold may vary. In summary, the dominant ROC curve and the top-ranked AUC of the Improved BiLSTM Network emphasize its superior efficacy in EEG-based depression recognition. This characteristic is particularly significant for an EEG depression classification system: a model that consistently attains high sensitivity and specificity (as demonstrated by a higher ROC curve) will be more trustworthy for clinical screening or diagnostic support, reducing the likelihood of missed detections of MDD and minimizing false alerts.

#### 4.2.4. Confusion Matrix Analysis

Figure 5 shows the confusion matrix of the improved bidirectional long short-term memory network. The matrix indicates that 10,317 HC epochs are correctly recognized as HC, while 1405 HC epochs are misclassified as MDD. For the MDD class, 7606 epochs are correctly identified, whereas 2550 MDD epochs are predicted as HC. These results reflect an asymmetric error profile, with the model exhibiting stronger discriminability for HC than for MDD.

From a clinical screening and decision-support perspective, a key advantage of the proposed model lies in its high specificity toward HC. Specifically, the HC false-positive rate is approximately 11.99% (1405/(10,317 + 1405)), corresponding to a specificity of 88.01%. This behavior suggests that the model is relatively conservative in assigning the MDD label and is less prone to incorrectly flagging healthy subjects as depressed, which is desirable for reducing unnecessary follow-up examinations and minimizing the burden caused by false alarms.

Meanwhile, when the model predicts the MDD class, it demonstrates high precision. The positive predictive value (PPV) for MDD reaches 84.41% (7606/(7606 + 1405)), indicating that the majority of MDD predictions are correct and that the decision boundary learned by the model yields a reliable positive output. Notably, this observation is consistent with the overall performance summary reported in Table 2, where the proposed method achieves 84.4% precision and 74.9% recall, implying that the confusion-matrix-derived class-wise statistics align with the metric-level evaluation.

In addition, the model achieves an MDD sensitivity of 74.89% (7606/(7606 + 2550)). Although false negatives (MDD→HC) remain non-negligible, the overall pattern suggests that the proposed architecture favors high-confidence MDD predictions while maintaining strong rejection capability for non-depressed cases. This trade-off is coherent with the design motivation of leveraging bidirectional temporal modeling and vector-based feature routing to enhance discriminative robustness under the adopted evaluation protocol.

#### 4.2.5. Ablation Experiment

**Figure 6:**
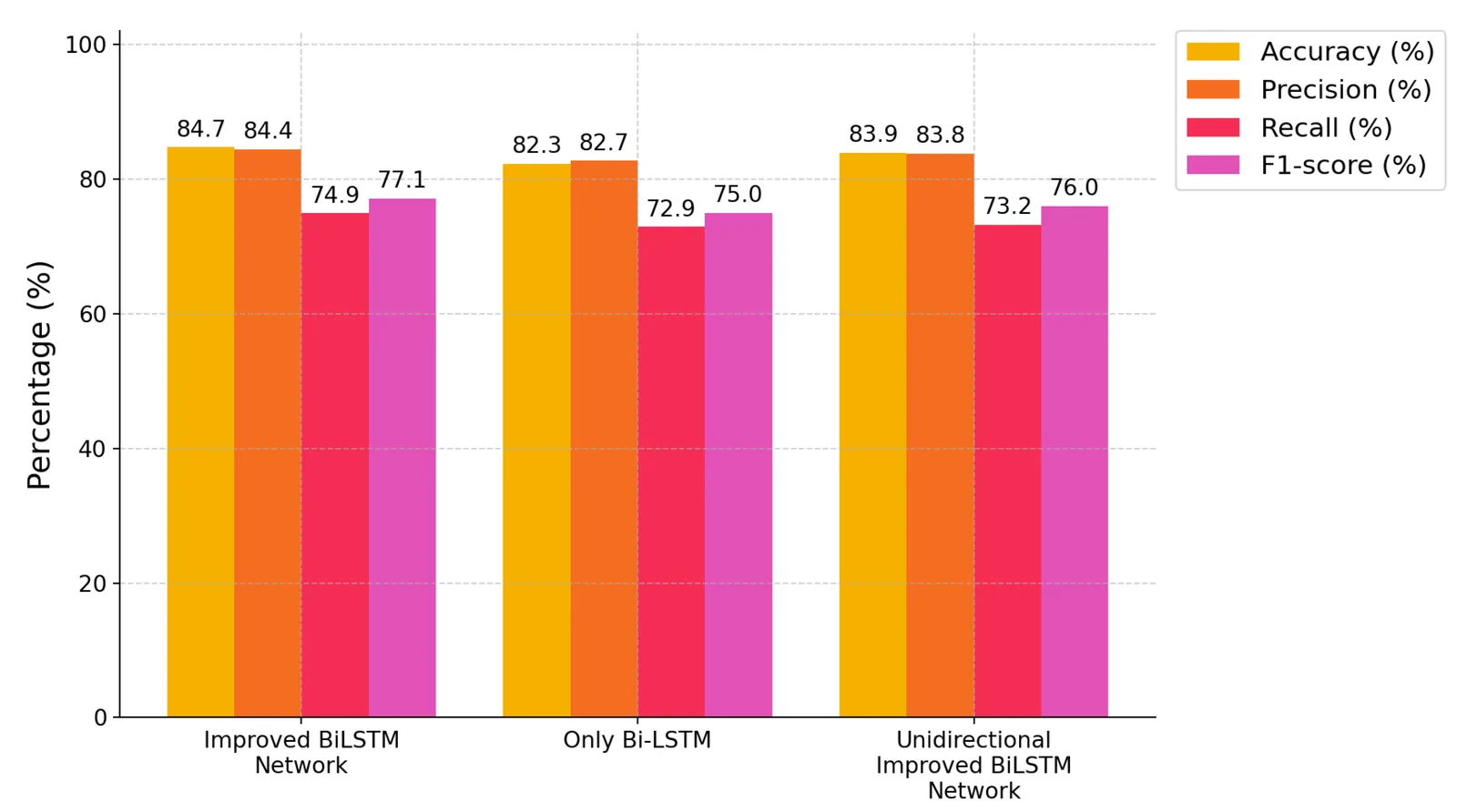
Ablation study comparison figure.

To analyze the contribution of each component in Improved BiLSTM Network, we conducted systematic ablation experiments, with the results summarized in Table 4. When the bidirectional LSTM was removed and only the Vector-Representation layer was used, all performance metrics showed a significant decline, confirming that modeling long-term temporal dependencies is crucial for EEG classification. Conversely, when the Vector-Representation layer was removed and only the bidirectional LSTM was used with a fully connected layer for classification, performance was also compromised, especially with noticeable drops in precision and F1-score, indicating that the Vector-Representation layer plays a key role in enhancing the model’s discriminative power and robustness. Additionally, replacing the bidirectional LSTM with a unidirectional LSTM led to a slight performance decline.The performance comparison of the ablation study is shown in the Figure 6.

#### 4.2.6. Frequency Band Experiment

To investigate the model’s performance across different frequency bands and the performance differences between combining and not combining frequency band data, we conducted a frequency band experiment. The five sub-bands include alpha, delta, theta, beta, and gamma, as well as the full band (fullband) and combined band (allbands). In this study, the gamma band specifically refers to the frequency range of 30–70 Hz, and frequency components above 70 Hz were not included in the analysis.

**Figure 7:**
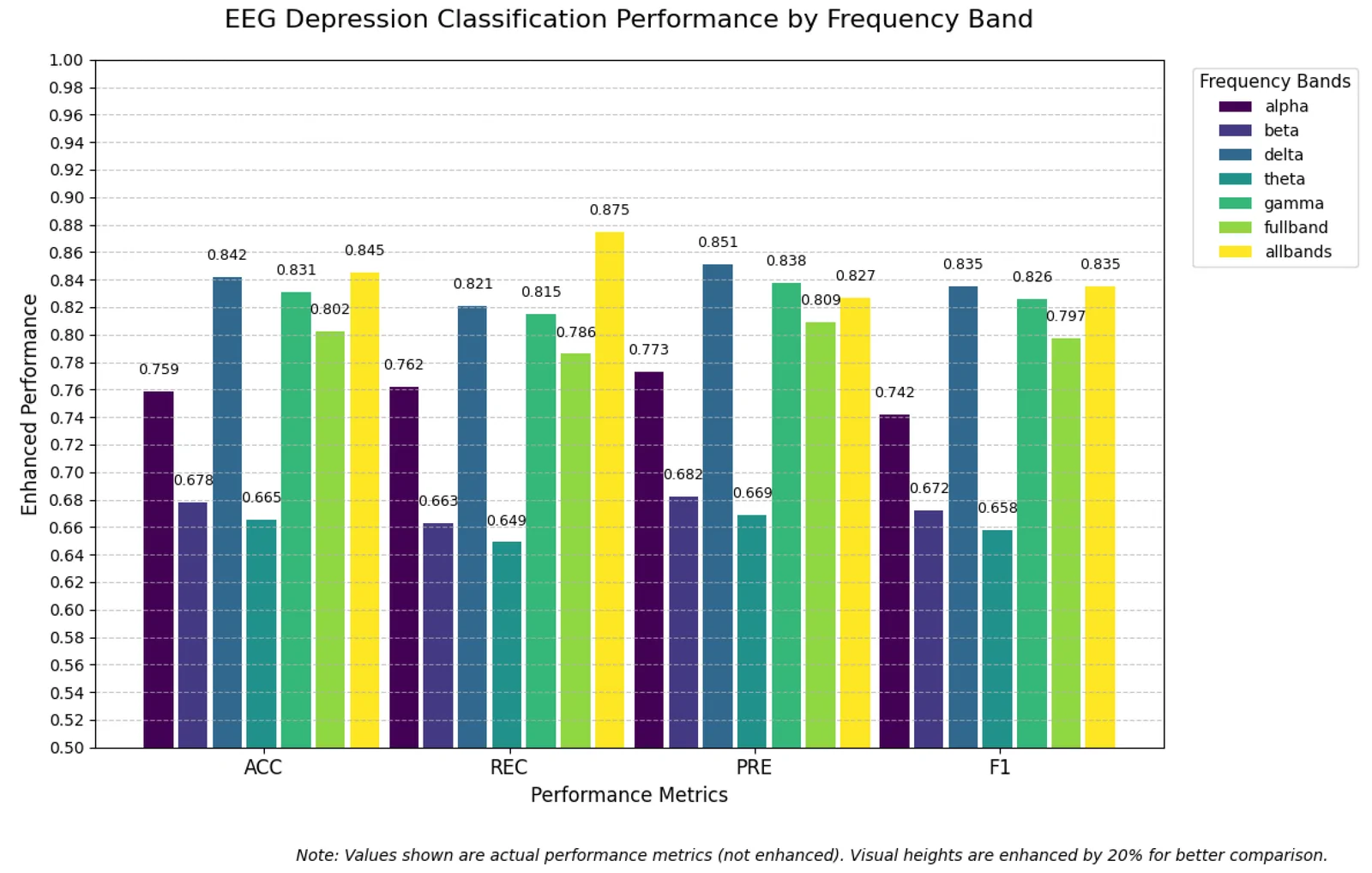
Performance comparison across different frequency bands and combined frequency bands.

The experimental results are shown in Figure 7. Comparing the single frequency bands, the model performs best in the delta band, followed by the gamma band, while performance in the alpha, theta, and beta bands is relatively lower. This indicates that MDD patients exhibit more differentiated features in the delta and gamma frequency bands compared to healthy controls, which may be related to the neurophysiological mechanisms of depression. Previous studies have reported that gamma power is associated with attention deficits in MDD patients [52], where the gamma activity typically lies within the low- to mid-gamma range (30–70 Hz), while delta oscillations are more prominent in pathological states caused by brain damage in conditions like depression [53]. Therefore, features in the delta and gamma bands may be more discriminative for MDD classification and provide beneficial biomarkers for MDD identification. Comparing single-band, full-band, and combined-band approaches, the combined band method achieved the best performance in key metrics like F1 and Accuracy, demonstrating that the proposed model, by using combined frequency bands, can extract more frequency domain features, thereby improving classification performance.

The Improved BiLSTM model’s efficacy was methodically confirmed in this experimental phase, which also suggested future paths for its advancement.

#### 4.2.7. Attention and Saliency Analysis

**Figure 8:**
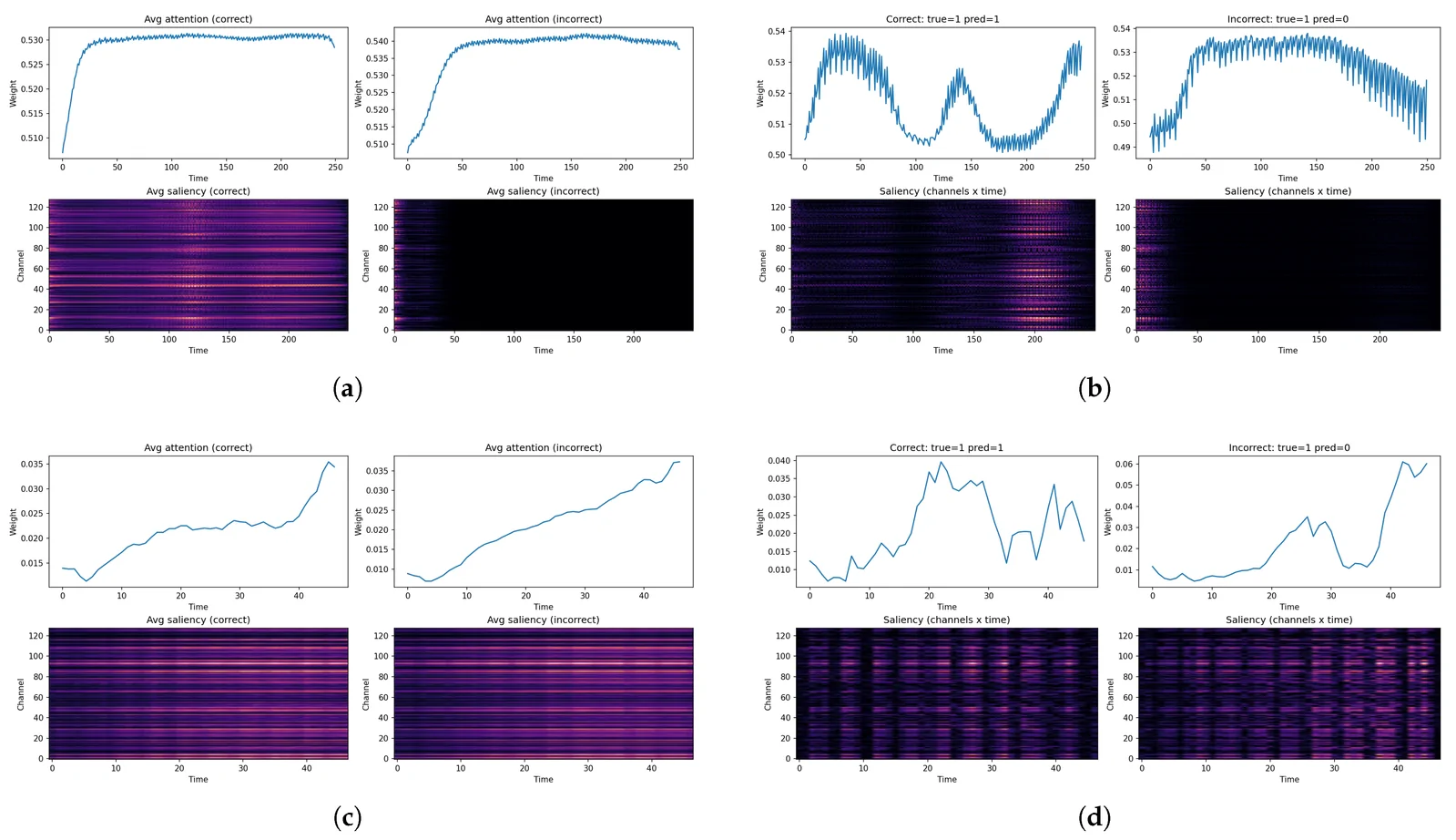
(**a**) The averaged attention curves and saliency heatmaps across all folds. (**b**) Representative examples from the second fold. (**c**) The averaged attention curves and saliency heatmaps across of CNN–LSTM baseline. (**d**) Representative examples from the second fold of CNN–LSTM baseline.

To further investigate the decision-making behavior of the proposed model, we conducted an in-depth analysis of the attention weight distributions and saliency maps under correct and incorrect predictions. Specifically, we compare (i) the averaged attention curves and saliency heatmaps across all folds as shown in Figure 8a, (ii) representative examples from the second fold as shown in Figure 8b, and further contrast these results with those obtained from the CNN–LSTM baseline, including its averaged results as shown in Figure 8c and second-fold examplesas shown in Figure 8d.

As shown in Figure 8a (top row), the Improved BiLSTM exhibits a clear difference in temporal attention behavior between correctly and incorrectly classified samples. For correct predictions, the average attention curve rises rapidly during the early time steps and then stabilizes at a relatively high and smooth plateau, indicating that the model is able to identify informative temporal segments early and maintain consistent focus throughout the sequence. In contrast, the attention curve corresponding to incorrect predictions is more irregular and exhibits larger fluctuations, without forming a stable plateau. This instability suggests that, when misclassification occurs, the model fails to establish a reliable temporal focus and instead distributes attention in a less structured manner.

A similar trend is observed in the CNN–LSTM baseline (Figure 8c, top row), but with notable differences. Although CNN–LSTM also shows increasing attention over time, the separation between correct and incorrect curves is less pronounced, and both curves tend to follow a more monotonic pattern. This indicates that CNN–LSTM relies more heavily on later temporal segments and lacks the early discriminative attention behavior observed in the Improved BiLSTM, which may partially explain its inferior classification performance.

The saliency heatmaps in Figure 8a (bottom row) further highlight the contrast between correct and incorrect predictions for the proposed model. For correctly classified samples, salient responses are broadly distributed across multiple channels and time steps, forming structured patterns rather than isolated activations. This suggests that the model integrates information from multiple electrodes and temporal segments in a coordinated manner. Conversely, the saliency map for incorrect predictions is markedly sparse, with most salient responses concentrated only in the very early time steps and rapidly vanishing thereafter. The absence of meaningful saliency in mid-to-late temporal regions implies that the model does not extract sufficiently discriminative features when making erroneous decisions.

In comparison, the CNN–LSTM saliency maps (Figure 8c, bottom row) show more uniform but weaker activations for both correct and incorrect cases. The spatial–temporal contrast between correct and incorrect predictions is less evident, indicating that CNN–LSTM may struggle to form sharp, class-specific feature representations across channels and time.

The above observations are further corroborated by the single-sample visualizations from the second fold (Figure 8b,d). In Figure 8b, a correctly classified sample by the Improved BiLSTM shows a well-defined attention peak and concentrated saliency regions aligned with high-activity EEG segments, whereas the incorrectly classified sample lacks clear attention peaks and exhibits diffuse or weak saliency across channels. By contrast, the CNN–LSTM examples in Figure 4 demonstrate relatively smoother but less discriminative attention profiles, and their saliency maps appear more homogeneous, even in incorrect cases, suggesting reduced sensitivity to subtle but critical EEG patterns.

Overall, these analyses indicate that correct predictions are associated with stable temporal attention and structured, distributed saliency, while incorrect predictions are characterized by unstable attention and sparse or ambiguous saliency patterns. This phenomenon suggests that misclassification is often linked to insufficiently confident or poorly localized feature attribution.

## 5. Discussion

### 5.1. Model Performance and Multi-Domain Fusion

The proposed Bidirectional temporal dependency modeling and vector routing aggregation mechanism model yielded superior performance on the MODMA EEG dataset compared to multiple baseline classifiers. It achieved 84.8% accuracy and an AUC of 0.88, outperforming all reproduced benchmarks. These results confirm the benefit of fusing temporal and spectral features in capturing complex depression-related EEG patterns. The model also showed a bias toward high specificity while maintaining a moderate sensitivity, reflecting a precision-oriented bias.

Experiments isolating individual EEG frequency ranges further revealed that delta and gamma bands were the most discriminative for depression (yielding the highest single-band accuracy), which aligns with reports of abnormal delta and gamma activity in MDD patients. Crucially, combining all five bands yielded the highest overall accuracy and F1-score, confirming that multi-band input provides complementary information beyond any single frequency band.

Ablation studies further demonstrated that each component of the architecture is indispensable. Removing the improved bidirectional LSTM module led to a marked drop in all metrics, underscoring the necessity of long-term temporal modeling. Likewise, replacing the vector-routing feature layer that preserves multi-channel configuration with a standard dense layer degraded performance, suggesting that preserving multi-channel configuration without pooling helps the model learn inter-channel dependencies implicitly Together, these findings highlight that the proposed spatiotemporal–frequency fusion design fully leverages the multi-domain EEG information to achieve its superior results.

To further interpret these results, we examined the model’s internal attention and saliency patterns on correctly classified instances. The attention mechanism in those cases exhibits a stable focus on relevant EEG segments, while the corresponding saliency maps display widely distributed activations across multiple channels and frequency bands. This stable, broad-based response indicates that the model’s predictions are driven by consistent multi-faceted EEG cues rather than any isolated feature, reinforcing its robust performance.

### 5.2. Comparison with Existing Approaches

Our framework also offers notable contrasts with other state-of-the-art EEG classifiers. Recent attention-based Transformer architectures for EEG emphasize global token interactions, treating EEG signals as sequences of tokens for self-attention. By contrast, our model preserves multi-channel configuration without pooling/downsampling; the model learns inter-channel dependencies implicitly across channels of electrodes across frequency bands and leverages a lightweight bidirectional LSTM to model temporal dynamics. This pooling/downsampling-free design avoids the spatial information loss caused by aggressive pooling and is computationally more efficient than Transformer-based schemes. Notably, it outperformed a self-attention CNN baseline in both accuracy and AUC, indicating that spatiotemporal–frequency fusion can rival more complex attention mechanisms on this task.

Furthermore, analysis of the attention and saliency patterns highlights differences between our Improved BiLSTM and a conventional CNN–LSTM baseline. The Improved BiLSTM can discriminate depressed from healthy EEG patterns at an earlier stage of the sequence and with greater spatio–temporal specificity, whereas the CNN–LSTM tends to require more accumulated evidence and exhibits a more diffuse focus across channels and time.

In addition, unlike complex multi-expert ensemble frameworks that combine specialized models for different contexts or emotional cues, we employ a single integrated model in a controlled resting-state scenario. Prior studies have proposed ensemble systems to handle varied interview or stimulus contexts, whereas we demonstrated that a single-model approach can effectively distinguish depressed versus healthy subjects using only resting-state EEG. The strong performance of our unified model suggests that elaborate ensembling may be unnecessary for depression detection. Overall, our design achieves a favorable balance between classification performance and implementation complexity, which is advantageous for practical EEG-based depression screening.

### 5.3. Limitations and Future Directions

Despite its promising results, this study has certain limitations. The evaluation was conducted on a single dataset (MODMA) with only 52 subjects (23 MDD patients and 29 healthy controls). While five-fold cross-validation mitigated overfitting, the model’s generalizability to independent cohorts or different EEG recording conditions remains unproven. Future studies should validate the approach on larger, more diverse datasets and assess cross-dataset robustness. The limited data also motivates exploring few-shot learning techniques to help the model adapt to new subjects or related tasks with minimal training examples. Addressing these data limitations is crucial for real-world clinical deployment, as the model must demonstrate reliable performance across diverse patient populations and recording conditions to be viable in practice.

Although we provide empirical proxy evidence for preprocessing robustness through window-length sensitivity and spectral band-pass evaluation, we did not conduct a full leave-one-step-out preprocessing ablation. A comprehensive ablation will be considered in future work, ideally with multi-site or cross-dataset validation where the impact of artifact-control and referencing choices can be assessed without compromising clinical validity.

Another limitation is the coarse labeling scheme of depressed vs. healthy, which may oversimplify the spectrum of depressive disorders. Depression severity and subtypes vary, and our model has not been evaluated on differentiating these nuances (e.g., moderate vs. severe depression or comorbid conditions). Future work should consider incorporating finer-grained labels or multi-class distinctions to increase the model’s clinical relevance. Additionally, our attention and saliency analysis of misclassified cases revealed model-specific shortcomings. In many error instances, the model’s attention was unstable or overly focused on EEG segments that appeared non-discriminative, and the corresponding saliency maps often highlighted noisy or irrelevant features. This suggests that when the model fails, it may be relying on spurious patterns, pointing to a need for further refinement. Future work could implement attention regularization techniques to encourage a more stable focus on meaningful signals, and incorporate a confidence-aware mechanism to detect and manage low-confidence predictions. Such extensions, along with broader validation, will determine whether the proposed multi-domain fusion approach generalizes across diverse conditions and captures subtle diagnostic differences.

## 6. Conclusions

This paper suggests an enhanced bidirectional LSTM model for EEG-based depression diagnosis. The model can automatically extract and integrate temporal, spatial, and spectral information from the EEG data by reconstructing multi-channel EEG signals into a multi-domain frame sequence and using a bidirectional LSTM network to learn temporal connections. Experiments conducted on a public dataset validate the effectiveness of the proposed method, with both accuracy and AUC significantly outperforming traditional approaches such as SVM, EEGNet, InceptionNet, Self-attention-CNN and conventional CNN-LSTM models. In addition, the use of combined multi-frequency inputs further enhances diagnostic performance, demonstrating the soundness of the multi-domain fusion design.

Although the proposed model achieves promising results in depression identification, its generalizability and applicability require further investigation. Future work may extend the evaluation to other psychiatric disorder datasets to examine cross-task generalization capability. Moreover, incorporating richer spatial-encoding mechanisms or attention-based modules could further improve the model’s ability to capture complex spatial patterns and enhance interpretability. Overall, this study provides a feasible and efficient technical framework for EEG-based automatic diagnosis of mental disorders and offers valuable insights for the application of multi-domain fusion deep learning methods in biomedical signal analysis.

## Figures and Tables

**Table 1:** Qualitative comparison with recent related works.

Ref.	Task	Input Representation	Key Idea
TNSRE 2023/2024 (B2-ViT) [11]	Seizure prediction	Multi-channel EEG tokens	Broad Transformer for global channel interactions
Sci Rep 2024 (TPRO-NET) [12]	EEG emotion recognition	DE + nonlinear enhanced features	Efficient Transformer encoder
BSPC 2025 (BPM + Bi-MGRU + Attention) [13]	Fatigue detection	2D power maps	Topographic map + recurrent + attention, hyperparameter optimization
TAFFC 2025 (MLM-EOE) [14]	Depression detection	Multimodal + emotion tags	Emotion labeling + expert ensemble
ACCTHPA 2025 [17]	Mental health diagnosis	Wavelet features	DWT + ML
IBRO 2024 [18]	Psychiatric disorders	PSD/FC features	Compare DL models (CNN-LSTM/BiLSTM etc.)
REEDCON 2023 [32]	Schizophrenia diagnosis	Time-series EEG	Bi-LSTM temporal modeling for efficient diagnosis

**Table 2:** Performance comparison of different models on the MODMA dataset.

Model	Acc (%)	Pre (%)	Rec (%)	F1 (%)
SVM [47]	70.7	67.2	62.1	64.1
EEGNet [49]	72.1	70.7	66.8	66.7
InceptionNet [48]	73.1	70.2	62.5	66.6
CNN-LSTM [39]	73.9	73.9	67.9	67.8
Self-attention-CNN [51]	82.9	83.6	72.8	77.7
Improved BiLSTM Network	84.7	84.4	74.9	77.1

**Table 3:** Performance under different hyperparameter configurations.

Hyperparameter	Values	Acc (%)	Pre (%)	Rec (%)	F1 (%)
BiLSTM hidden size	32	82.9	82.1	71.8	75.6
	64	84.7	84.4	74.9	77.1
	128	84.9	84.1	74.0	76.6
Vector-Representation Layer	8	83.6	83.0	73.1	76.9
	16	84.7	84.4	74.9	77.1
	32	84.5	83.9	75.0	77.0
Routing iterations	1	83.4	82.8	72.6	75.7
	3	84.7	84.4	74.9	77.1
	5	84.6	84.0	75.1	77.1
Window length (s)	1	83.1	82.6	72.3	75.4
	2	84.7	84.4	74.9	77.1
	4	84.9	83.7	74.1	76.7

**Table 4:** Ablation Experiment Results.

Model	Acc (%)	Pre (%)	Rec (%)	F1 (%)
Improved BiLSTM Network	84.7	84.4	74.9	77.1
Only Vector-Routing Layer	80.1	79.9	71.5	71
Only Bi-LSTM	82.3	82.7	72.9	75
Unidirectional Improved BiLSTM Network	83.9	83.8	73.2	76.0

## Data Availability

Publicly available data were used for this work. The dataset can be found at “Cai, H.MODMA dataset: a Multi-modal Open Dataset for Mental-disorder Analysis” https://modma.lzu.edu.cn/data/application/, accessed on 25 November 2024.
